# The serine/threonine kinase 33 is present and expressed in palaeognath birds but has become a unitary pseudogene in neognaths about 100 million years ago

**DOI:** 10.1186/s12864-015-1769-9

**Published:** 2015-07-22

**Authors:** Tobias Lautwein, Steffen Lerch, Daniel Schäfer, Erwin R. Schmidt

**Affiliations:** Institute for Molecular Genetics, Johannes Gutenberg University Mainz, Johann-Joachim-Becherweg 32, 55128 Mainz, Germany; Departement of Neurology, University Medical Center, Johannes Gutenberg-University Mainz, Langenbeckstr.1, 55131 Mainz, Germany; Departement of Pediatric Oncology, Hematology and Clinical Immunology, University Children’s Hospital, Medical Faculty, Heinrich Heine University, Moorenstr. 5, 40225 Düsseldorf, Germany

**Keywords:** Serine/threonine kinase 33, Aves, Pseudogene, Evolution, Genetic redundancy, Non-orthologous gene displacement

## Abstract

**Background:**

Serine/threonine kinase 33 (STK33) has been shown to be conserved across all major vertebrate classes including reptiles, mammals, amphibians and fish, suggesting its importance within vertebrates. It has been shown to phosphorylate vimentin and might play a role in spermatogenesis and organ ontogenesis. In this study we analyzed the genomic locus and expression of *stk33* in the class Aves, using a combination of large scale next generation sequencing data analysis and traditional PCR.

**Results:**

Within the subclass Palaeognathae we analyzed the white-throated tinamou (*Tinamus guttatus*), the African ostrich (*Struthio camelus*) and the emu (*Dromaius novaehollandiae*). For the African ostrich we were able to generate a 62,778 bp long genomic contig and an mRNA sequence that encodes a protein showing highly significant similarity to STK33 proteins from other vertebrates. The emu has been shown to encode and transcribe a functional STK33 as well. For the white-throated tinamou we were able to identify 13 exons by sequence comparison encoding a protein similar to STK33 as well.

In contrast, in all 28 neognath birds analyzed, we could not find evidence for the existence of a functional copy of *stk33* or its expression. In the genomes of these 28 bird species, we found only remnants of the *stk33* locus carrying several large genomic deletions, leading to the loss of multiple exons. The remaining exons have acquired various indels and premature stop codons.

**Conclusions:**

We were able to elucidate and describe the genomic structure and the transcription of a functional *stk33* gene within the subclass Palaeognathae, but we could only find degenerate remnants of *stk33* in all neognath birds analyzed. This led us to the conclusion that *stk33* became a unitary pseudogene in the evolutionary history of the class Aves at the paleognath-neognath branch point during the late cretaceous period about 100 million years ago. We hypothesize that the pseudogenization of *stk33* might have become fixed in neognaths due to either genetic redundancy or a non-orthologous gene displacement and present potential candidate genes for such an incident.

**Electronic supplementary material:**

The online version of this article (doi:10.1186/s12864-015-1769-9) contains supplementary material, which is available to authorized users.

## Background

Serine/threonine kinase 33 (*stk33*) was first discovered in the course of comparative genome analyses of human chromosome 11p15.3 and its orthologous region in the mouse [1–3]. This region is highly syntenic (see Additional file 1: Figure S1) and known to be associated with several diseases including different types of cancer [4–8]. *Stk33* is predominantly expressed in testis, certain brain regions and embryonic organs such as brain, heart and spinal cord in human and mouse, implying that it might be involved in spermatogenesis and organ ontogenesis [9]. Its protein product has been classified as a member of the calcium/calmodulin-dependent kinase group [1]. STK33 has been shown to co-localize with the intermediate filament protein vimentin in tanycytes of mouse, rat and hamster [10] and kinase assays demonstrated that STK33 is able to phosphorylate vimentin *in vitro* [11]. STK33 and vimentin are associated *in vivo* and this interaction is not mediated by any additional protein as demonstrated by co-immunoprecipitation and co-sedimentation assays [11]. Thus, STK33 might be involved in the dynamics of the intermediate filament cytoskeleton by phosphorylating vimentin [11]. Furthermore, *stk33* has attracted attention since it has been identified as an essential component for the survival of KRAS-dependent cancer cells by high-throughput RNA interference (RNAi) screens [12–14]. It has also been associated with pancreatic cancer [15]. However, the role of *stk33* in KRAS-dependent cancer cells remains disputed because other research groups were not able to find a correlation between the knockdown or inhibition of *stk33* and KRAS-dependent cancer cell survival, neither using RNAi nor small molecule inhibitors [16–19]. Nevertheless, recent studies found an association of *stk33* with colorectal cancer [20], hepatocellular carcinoma [21], hypopharyngeal squamous cell carcinoma [22] and lung cancer [23].

To date, *stk33* has been found only in the subphylum Vertebrata. So far it has been found in multiple vertebrate classes including mammals, reptiles, amphibians and fish, but not in birds (A phylogeny of all known STK33 proteins has been built, see Additional file 1: Figure S3). Neither the analyses of the International Chicken Genome Sequencing consortium [24], nor our own screening of the chicken (*Gallus gallus*) genome for *stk33* revealed a functional copy (see below). So far, this was the only vertebrate class with no indication for a functional *stk33*. Since *stk33* genes are present as well in mammalians as in phylogenetically older vertebrate species (see Additional file 1: Figure S3), we wanted to know whether birds do not have *stk33* genes in general or if the chicken is an exception. Therefore, we analyzed the genomes and transcriptomes of more than 30 different bird species using deep sequencing and publicly available next generation sequencing data [25].

The class of Aves comprises about 10,500 extant species [26] and the majority of these species arose from a rapid radiation after a mass extinction event about 66 million years ago within neonaves [27, 28]. It is divided into the clade Palaeognathae, including the flightless ratites and the tinamous, and the clade Neognathae, including all other extant bird species [27, 29, 30].

In this article, we report that *stk33* is present and expressed in paleognath birds, but has become a unitary pseudogene within the subclass Neognathae during the cretaceous period about 100 million years ago.

## Results

First, we analyzed the chicken (*Gallus gallus*) genome for the presence of a functional copy of *stk33*. In human (*Homo sapiens*) and mouse (*Mus musculus*) *stk33* is located in a highly syntenic region on chromosome 11p15.3 and chromosome 7E3, respectively [2]. In the chicken this region could be identified on chromosome 5, whereas in the chicken no *stk33* has been annotated in this region (see Additional file 1: Figure S1). Therefore we screened the whole genome and this region in particular for the presence of *stk33*. These genomic screens revealed the presence of only eight remnant exons containing both frameshifting indels and multiple stop codons (Fig. 3) in this region and multiple large genomic deletions that have led to the deletion of several exons. Extensive BLASTn searches of the chicken genome did not reveal a duplication or a retrotransposition event for *stk33*.

This led us to the conclusion that *stk33* has become a unitary pseudogene in the chicken, raising the question if the chicken is an exception or if *stk33* is missing in birds in general.

### *Stk33* is present and expressed in the subclass Palaeognathae

**Fig. 1:**
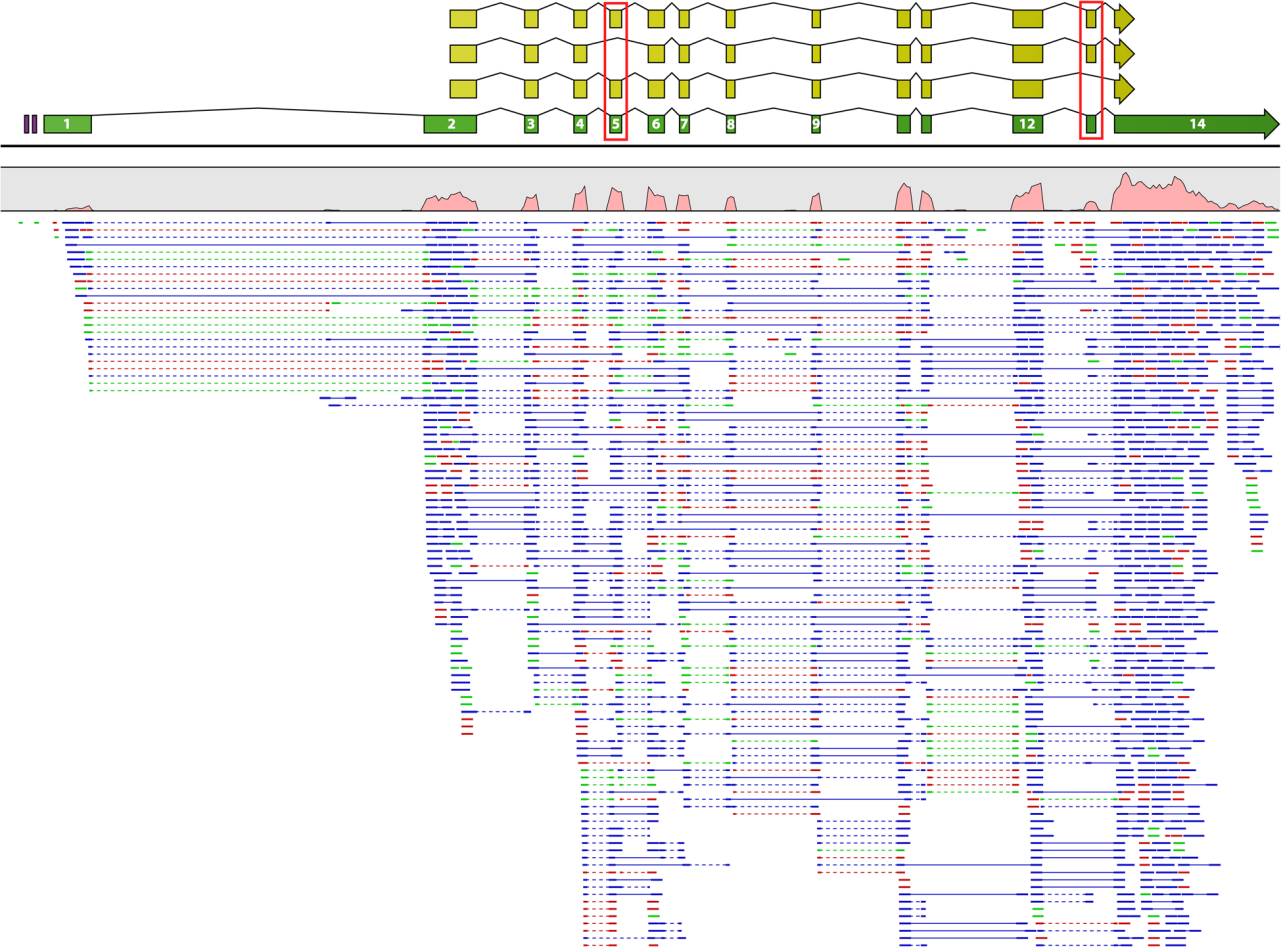
Stk33 gene structure in ostrich. Transcriptomic reads obtained from total RNA of the ostrich cerebellum and testis were mapped back to the genomic reference. Paired-end reads mapping within their paired distance are shown in blue connected by a blue solid line, forward reads in green and reverse reads in red. Broken lines depict split reads. The read coverage is illustrated as a red graph. The *stk33* mRNA is annotated as a green arrow, the corresponding coding sequences as yellow arrows. Purple rectangles show GC boxes. All introns are shown with 10 % of their original size

To answer this question, we first reconstructed the *stk33* genomic locus and the intron-exon structure in the most basal extant bird, the African ostrich (*Struthio camelus*) which belongs to the subclass Palaeognathae. Illumina sequencing of total RNA generated from both cerebellum and testis yielded 102,624,190 reads (Table 2). Since no genomic data was available as a reference at the time, we performed de novo assemblies of the Illumina reads using k-mer sizes between 20 and 64. These assemblies yielded between 42,726 and 67,174 contigs with an average length between 781 and 1010 bp. All contigs from all assemblies were analyzed by BLASTn searches against the non-redundant nucleotide collection (nr/nt) database. A total of 16 assemblies contained *stk33* relevant contigs, which were assembled into a single scaffold of 4,020 bp length. For a preliminary annotation of exons on this scaffold the publicly available *stk33* sequence from the green anole (*Anolis carolinensis*) [GenBank:XM_008117649] was used. These exons were verified by RT-PCR and the intron-exon boundaries were determined using intron-spanning PCRs on genomic DNA followed by Sanger sequencing. We obtained the full length mRNA sequence and identified two alternative transcriptional start sites as well as two different polyadenylation sites by RACE PCR. In summary, a *stk33* mRNA sequence of 3,366 bp with a total of 14 exons was established (Fig. 1), all flanked by consensus splice sites (GT/AG). For the genomic structure of *stk33* all introns and the promoter region were amplified by PCR. Finally a genomic contig of 62,778 bp was assembled [GenBank:KP072780]. In the promoter region, two GC-box elements were identified at positions −43 and -55. For verification, the Illumina transcriptome reads were mapped back to the genomic reference contig (Fig. 1).

**Fig. 2:**
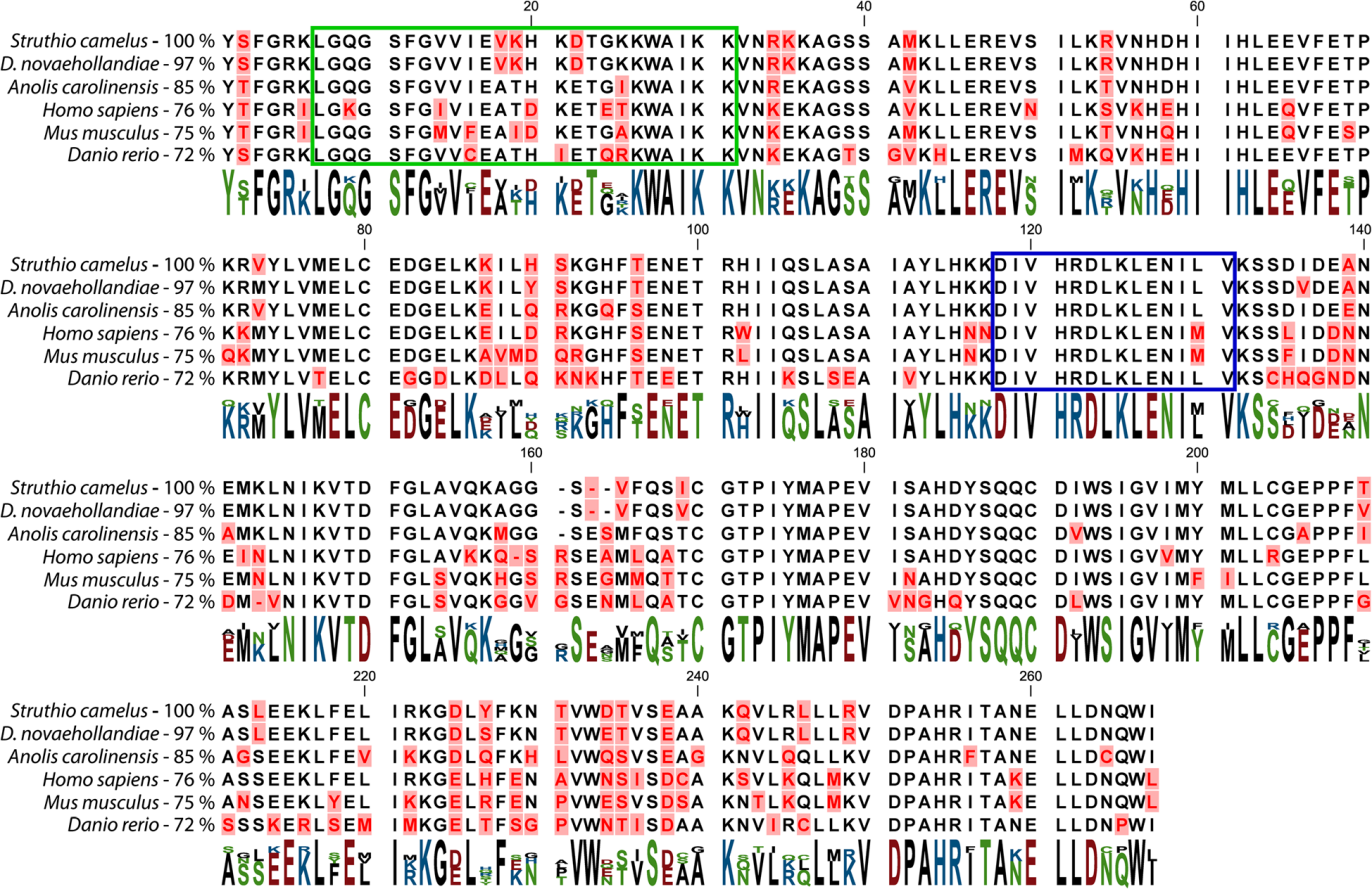
Amino acid sequence alignment of the STK33 catalytic domains from different species. Percentage of sequence identities corresponding to the catalytic domain are shown next to the corresponding sequences. The ATP binding site is framed by a green rectangle and the active site by a blue rectangle. Mismatches are emphasized in red

The ostrich *stk33* mRNA sequence contains a single open reading frame (ORF) of 1,635 bp encoding a 544 amino acid protein of 59.9 kDa that shows highly significant (E = 0.0) similarity to STK33 of the American alligator (*Alligator mississippiensis*), green sea turtle (*Chelonia mydas*), Chinese softshell turtle (*Pelodiscus sinensis*) and other reptiles. Protein analysis employing InterPro [31] showed the presence of a serine/threonine protein kinase catalytic domain, an ATP binding site and an active site, suggesting that all features of an active kinase are present (Fig. 2). The ostrich STK33 catalytic domain shows high similarity to STK33 proteins from other species (Fig. 2). A GC-rich region of 241 bp was found at the 5′ end of the transcribed region with a GC content of 81 % that seems to be unique for the ostrich (Fig. 1). Mappings of the transcriptomic data to the genomic reference contig revealed two alternative splice variants, skipping either exon 5 or exon 13 (Fig. 1). All splice variants were verified by PCR using splice variant specific primers. The existence of a splice variant lacking both exon 5 and exon 13 was precluded by PCR. The splice variant lacking exon 13 shows no change within the functional domains, whereas the splice variant lacking exon 5 contains a by 35 aa shortened serine/threonine kinase catalytic domain. However both the active site and the ATP binding site are not affected in both splice variants.

Encouraged by our findings that *stk33* is expressed in the African ostrich, we investigated if the genomes of other paleognath birds contain a functional *stk33* gene. For this purpose we chose the closely related emu (*Dromaius novaehollandiae*) and the white-throated-tinamou (*Tinamus guttatus*). For the emu we used transcriptomic Illumina data of the embryonic brain with a total of 51.1 Mio reads (Illumina) and 182,922 reads (454) from adult brain (Table 2). Since the publicly available genomic data [SRA: SRP019803] was of insufficient quality for the generation of a suitable reference sequence, we mapped the emu transcriptomic reads to the ostrich *stk33* mRNA sequence and extracted the consensus sequence. The transcriptomic reads were mapped back to this consensus sequence for verification. This led to an mRNA sequence of 2,560 bp in length split into 14 exons. The mRNA sequence contains a single open reading frame of 1,635 bp which shows 94 % identity (E = 0.0) with the ostrich mRNA sequence. The emu ORF encodes a 544 aa protein of 60.1 kDa. Amino acid sequence alignments of the ostrich and emu STK33 proteins show 97 % identity (E = 0.0) within the catalytic domain (Fig. 2). Across the whole protein sequence the similarity is 91 %. The emu STK33 also contains a serine/threonine protein kinase catalytic domain, an ATP binding site and an active site.

**Fig. 3:**
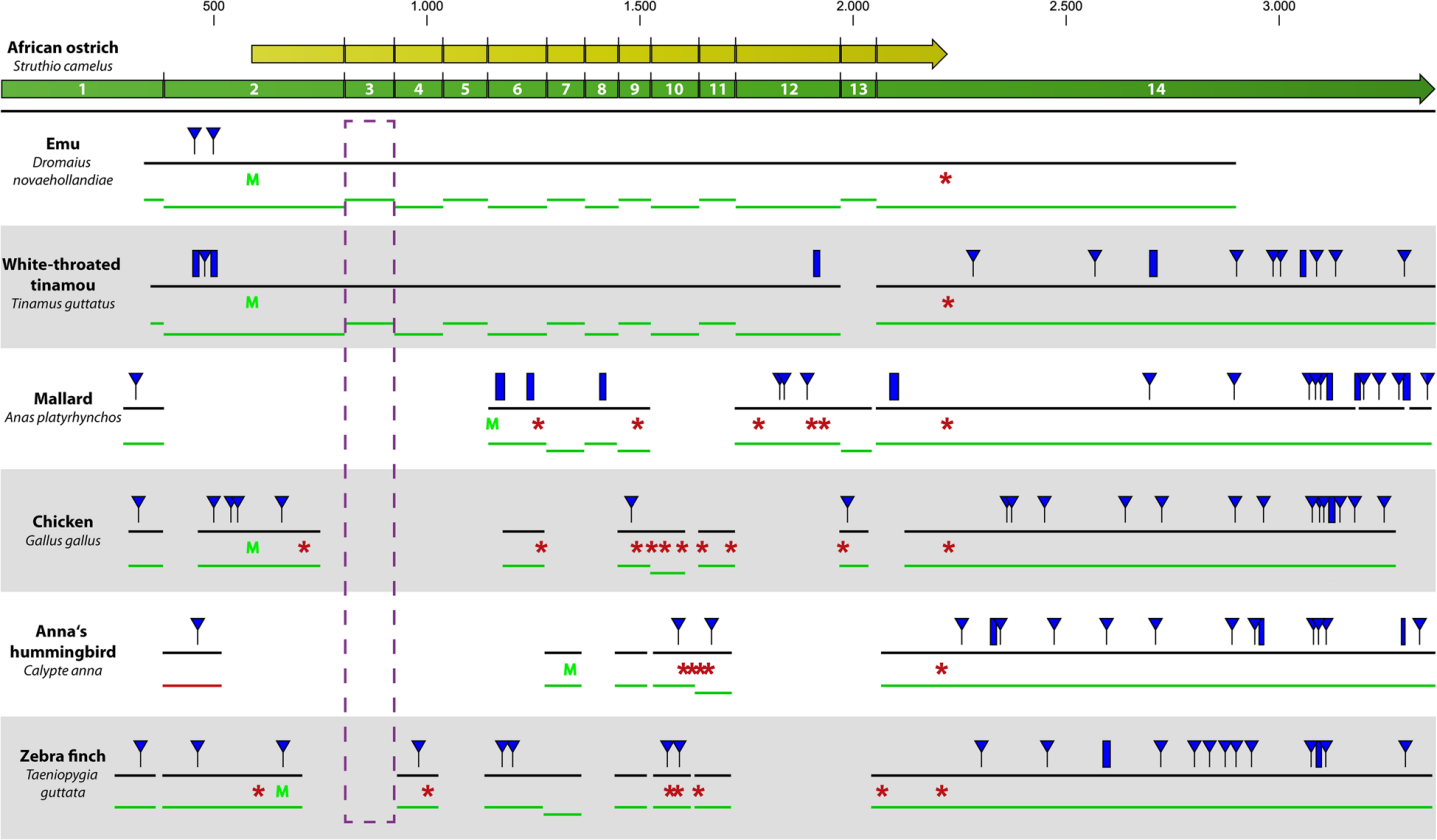
Sequence alignments of identified *stk33* exons of different birds to African ostrich *stk33* mRNA. The ostrich *stk33* mRNA is shown as a green arrow, the open reading frame as a yellow arrow. Green lines indicate the mapped exons, red lines indicate inversed exons. Small indels (<5 bp) are shown as blue triangles, large indels (>5 bp) as blue rectangles. Stop codons are illustrated as red asterisks and start codons as a green capital “M”. Black lines depict consensus sequences. The exon 3 region is highlighted by purple broken lines

For the white-throated-tinamou we focused on the genomic analysis, since no transcriptomic SRA data is available. This analysis showed that the white-throated-tinamou *stk33* contains only 13 Exons instead of the 14 seen in the African ostrich and emu, due to a large genomic deletion of 2.5 kb, including exon 13 [GenBank:BK008887]. However, the deletion of exon 13 does not interrupt the open reading frame and there are no indels or point mutations present elsewhere in the coding region, that would lead to frameshifts or premature stop codons. The same applies to a 6 bp deletion within exon 12 (Fig. 3). Furthermore, the African ostrich is expressing a splice variant lacking exon 13, which is not crucial for the function of STK33 as a kinase, because this exon is not part of the serine/threonine kinase catalytic domain. In summary, we can conclude that *stk33* is present and expressed in all birds of the subclass of Palaeognathae.

### *Stk33* has become a unitary pseudogene in Neognathae

Since we had evidence that a functional copy of *stk33* is missing in the genome of the chicken, we wanted to know whether this is a particular case for galliformes or whether this is generally the case for all neognath birds. Therefore, we examined a total of 28 neognaths (Additional file 1: Table S2) on a genomic level and four on a transcriptional level in order to achieve a good sample distribution over the Neognathae pedigree (see Additional file 1: Figure S2) [29, 27, 30]. The genomic studies were carried out as outlined for the white-throated-tinamou.

All of the 28 neognaths analyzed showed only remnants of non-functional *stk33* sequences. Frequent large genomic deletions of several kilobases within the *stk33* locus were observed, leading to the loss of several exons. BLASTx searches of the remnant exons showed multiple frameshifting indels and frequent SNPs, leading to the generation of multiple premature stop codons within the *stk33* ORF (Fig. 3 and Additional file 2: Figure S5). Even the closest relative to the paleognath birds in our list, the mallard (*Anas platyrhynchos*) shows a severely degenerated *stk33*, including the deletion of six exons, several indels and multiple premature stop codons (Fig. 3). In the Anna’s hummingbird (*Calypte anna*) an additional genomic inversion of a 3 kb fragment of the *stk33* gene is present, leading to the inversion of the 5′ end of exon 2 (Fig. 3). All birds examined belonging to the order Passeriformes (golden-collared manakin, American crow, Tibetan ground tit, collared flycatcher, zebra finch, white-throated sparrow, medium ground finch), which represents about 60 % of all extant avian species, showed deletions of the exons 3, 5, 8, 12 and 13, several indels and premature stop codons (see Fig. 3 and Additional file 1: Figure S1). All other neognaths show similar severe mutations (see Additional file 1: Figure S1). Interestingly, all neognaths examined share the deletion of exon 3.

We have investigated whether *stk33* is still transcribed in spite of the many mutations found in the neognath *stk33* genes. We also looked for the possibility that in neognaths *stk33* might have potentially acquired new exons in the course of evolution and thus, a functional variant of *stk33* might have been restored. For this analysis, we downloaded several transcriptomic SRA data sets (Table 2) of tissues in which transcription of *stk33* has been described previously [9, 10]. The data included more than 1.5 Billion reads from chicken (*Gallus gallus*), about 200 million reads from the white-throated sparrow (*Zonotrichia albicollis*) and 50 million reads from the collared flycatcher (*Ficedula albicollis*). In addition, we sequenced cDNA generated from zebra finch (*Taeniopygia guttata*) brain, yielding 9.5 million reads (Table 2). These data sets were then mapped to the appropriate genomic *stk33* target region and to the *stk33* mRNA sequence from the African ostrich. In all this data we could not detect any *stk33*-related sequences. Furthermore, RT-PCR from cDNA using pairs of primers designed to the remnant exon sequences did not yield any products.

In order to exclude that a duplication or a retrotransposition of *stk33* occurred during the course of evolution, we performed both transcriptional de novo assemblies and extensive BLASTn searches of the WGS assemblies. These analyses did not reveal any duplicated or retrotransposed *stk33* copies elsewhere in the genome, so that we can conclude that *stk33* turned into a unitary pseudogene in neognaths and is no longer expressed.

## Discussion

In this study we used a combination of large scale next generation sequencing data analysis and PCR to elucidate the genomic structure and the transcription of the *stk33* gene in the phylogenetic class of Aves. In both ostrich and emu, belonging to the phylogenetically older Palaeognathae, we were able to prove the presence of a functional *stk33* which is also undoubtedly transcribed. In the third paleognath, the white-throated tinamou, genomic analysis showed the presence of 13 exons with no frameshifting indels or premature stop codons within the coding region. We therefore conclude that the white-throated tinamou, as ostrich and emu, carries a functional copy of *stk33*. In the future this will need to be proven by transcriptomic and/or proteomic analyses. In summary, we can conclude that *stk33* is present and expressed in all paleognath birds.

In neognaths we found only non-functional remnants of *stk33*. There were no transcripts of these *stk33* remnants detectable. Genomic deletions, indels and premature stop codons clearly led to non-functionality of the degenerate *stk33* remnants (Fig. 3 and Additional file 2: Figure S5). Furthermore, there is neither evidence for a functional *stk33* copy elsewhere in the genome nor are new exons acquired to compensate for those that have been lost. This leads us to the conclusion that *stk33* has become a pseudogene in neognaths during the evolutionary history of the class Aves. Although there is ample evidence that no gene duplication occurred, a copy of *stk33* could have been retrotransposed back into the genome and somehow become a functional substitute for the original *stk33*. But since these so called processed pseudogenes are generally considered to be “dead on arrival” [32, 33], due to the lack of a promoter, this scenario would be considered highly unlikely. In addition, all attempts to identify any copies of *stk33* elsewhere in any neognath genome failed, so that we are rather confident that this subclass of birds is the only one among vertebrates lacking *stk33,* as can be inferred from those vertebrates with a present STK33 (see Additional file 1: Figure S3). Such gene loss is a common and important evolutionary process and has been described in bacteria [34], eukaryotes [35] and archaea [36]. It is very abundant in some lineages such as bacteria or tunicates, that have undergone considerable genome reduction during their evolution [34, 37–40]. Birds also have undergone gene loss and genome reduction compared to other tetrapod classes, but not to the same extent as in bacteria or tunicates [41–43].

It is interesting that all neognaths lack exon 3, indicating that this chromosomal deletion may have been the initial mutation leading to the degeneration of *stk33*. This mutation most likely occurred at a very early stage in the diversification of the class of Aves during the late cretaceous period about 100 million years ago, at the branching point of Palaeognathae and Neognathae (see Additional file 1: Figure S2) [27, 29, 30]. Interestingly, a current large scale comparative genomics study in birds could show that the origin of neognath birds was accompanied by an elevated rate of genomic rearrangements [30]. This and the long time span are in accordance with the drastic changes seen in the *stk33* locus in neognaths. There are cases where pseudogenes have gained new functions expressing non-coding RNAs that can have a variety of different regulatory functions [44]. However, in spite of intensive searches we could not detect any *stk33* related transcripts in any of the neognath bird tissues analyzed. Therefore, a function of the *stk33* pseudogene is highly unlikely. This leads to the question, which gene could have taken over the function of *stk33* in neognaths or if its function has become dispensable.

In the following we would like to discuss different hypothesis about how the loss of *stk33* could have been tolerated and become fixed in neognath birds. One possibility could be, that the function of the *stk33* gene could have become dispensable, leading to the fixation of the pseudogene by genetic drift [45]. We believe this to be unlikely since *stk33* has been implicated in critical functions such as spermatogenesis and organogenesis [9] and there is no obvious indication to assume that neognath birds can manage without these functions of *stk33* whereas paleognath birds and other vertebrates cannot. Following this assumption, *stk33* could have undergone subfunctionalization, a process where a pair of genes that resulted from a duplication event retained different subfunctions of the original gene [46, 47]. If one of these subfunctions should have become dispensable, the corresponding paralogous gene would have acquired the observed deleterious mutations. But since we could not find any evidence for a *stk33* duplication event in neognath birds, we can preclude this possibility.

A third possibility is given by the “less is more” hypothesis. It states that gene loss can be an advantageous event and thus can serve as an engine of evolutionary change [48, 49]. Even though this may seem contradictory, since it suggests evolutionary innovation by degeneration, there are several examples of such a positive selection for gene loss [50–52]. One could argue that the loss of *stk33* may be beneficial, since it has been identified as an essential component in several types of cancer [12–15, 20–23]. However, the role of *stk33* in cancer remains controversial [16–19] and we do not have any indication for a beneficial effect due to the lack of *stk33*, making this a rather unlikely hypothesis. Nevertheless, analyzing if neognaths display any form of immunity to these types of cancer could represent an interesting approach, since they constitute natural knockouts for *stk33*.

Another possible explanation for the toleration of gene loss is the phenomenon of genetic redundancy, which can be caused by either gene duplication or convergent evolutionary processes, leading to proteins that are performing the same function, but are unrelated in sequence [53, 54]. Due to the lack of selective pressure on these genes, genetic redundancy would be considered to be evolutionary unstable. However, genetic redundancy appears to be quite frequent in genomes of higher organisms, implying that redundant genes can be evolutionary stable under certain conditions [54–58]. One model describing a stable genetic redundancy takes pleiotropy into consideration. If gene A performs function f1 and gene B performs both functions f1 and f2, but f1 with a slightly reduced efficacy, this genetic redundancy would be considered as evolutionary stable [59]. The presence of different splice variants in ostrich, human and mouse [1] strongly indicates that STK33 has more than one function and that vimentin may not be its only substrate [11]. This is supported by the fact that STK33 can also be found in vimentin-negative cells [11]. If we presume *stk33* to be gene B and assume that the function f2 has become irrelevant to the neognath lifestyle, *stk33* would have inevitably been lost in neognath birds.

A variant of the latter possibility is that *stk33* may have become dispensable, because it has been functionally substituted by a non-orthologous, but functionally analogous kinase in neognaths, a process called non-orthologous gene displacement (NOD). Several examples of such a NOD have been reported [60–64].

Regardless of whether genetic redundancy or non-orthologous gene displacement is causal for the pseudogenization of *stk33* in neognath birds, a second kinase that is able to phosphorylate vimentin has to be involved. For this, only kinases that are able to phosphorylate vimentin in the same regions as STK33 are considered as candidates. So far, the exact phosphorylation sites of STK33 have not yet been determined, but they have been circumscribed to at least one phosphorylation site between amino acids 30 and 42 and at least a second phosphorylation site located between amino acids 50 and 80 within the amino-terminal non-α-helical head domain by kinase assays using truncated vimentin mutants [11]. A total of ten kinases has been shown to phosphorylate vimentin (see Table 1) [65–80]. All these kinases are present in birds [41]. Since vimentin could be regulated differently in neognaths or birds in general, an alignment of the vimentin amino-terminal head domain has been built. This alignment shows that most of the phosphorylation sites are conserved between human, mouse and birds (Additional file 1: Figure S4). This suggests that vimentin is regulated in a similar way compared to human and mouse.

**Table 1:** Known vimentin phosphorylating kinases and their phosphorylation sites

Kinase	Site(s)	References
Rho-kinase	Ser-38; Ser-71	[65]
Protein kinase C	Ser-33; Ser-50	[66, 67]
CaMKII	Ser-38; Ser-82	[68, 69]
Protein kinase A	Ser-38; Ser-72	[70]
MAPKAP kinase-2	Ser-38; Ser-50; Ser-55; Ser-82	[71, 72]
Aurora kinase B	Ser-72	[73]
CDK1	Ser-55	[74–76]
Pak1	Ser-55	[77, 78]
Plk1	Ser-82	[79]
p37	Ser-457; Ser-458	[80]

The phosphorylation sites of two kinases, Plk1 and p37, are located outside the amino-terminal head domain [79, 80] and thus probably are no candidates that could substitute *stk33*. Three other kinases (Pak1, CDK1, Aurora kinase B) are also not suitable candidates since they only have a single phosphorylation site [73–78]. Even though MAPKAP kinase 2 and CAMKII have at least one phosphorylation site within the same region that is phosphorylated by STK33, they both also phosphorylate Ser-82 [68, 69, 71, 72], making them unlikely substituents for STK33. The three remaining kinases, Rho-kinase, protein kinase C and protein kinase A all have 2 phosphorylation sites within the same region as STK33 (Table 1) [65–67, 70]. However the vimentin alignment clearly shows that Ser-50, which is phosphorylated by protein kinase C [66, 67], is not conserved in birds (Additional file 1: Figure S4), making it a rather unlikely candidate. Thus Rho-kinase and protein kinase A represent the best candidates to compensate for the loss of *stk33* in neognath birds, since their phosphorylation sites are within the same region as those for *stk33* and are highly conserved in birds (Additional file 1: Figure S4).

Further studies about *stk33* on the genomic and protein level, especially about the exact phosphorylation sites of STK33, are necessary to further elucidate its function and to give additional indications towards the actual substituent. Without further knowledge all hypothesis remain speculative.

## Conclusions

Here, we elucidate and describe the genomic structure and the transcription of the serine/threonine kinase 33 (*stk33*) in paleognath birds, such as the African ostrich (*Struthio camelus*), emu (*Dromaius novaehollandiae*) and the white-throated tinamou (*Tinamus guttatus*) using both large scale next generation sequencing data analysis and PCR. We were able to show that all three paleognath birds analyzed carry functional and expressed copies of *stk33* and thus, we concluded that *stk33* is present and expressed in paleognath birds in general, as it is the case in all other vertebrate classes.

Surprisingly, in neognaths birds we found only degenerate remnants of *stk33* that have become non-functional by genomic deletions, indels and premature stop codons (Fig. 3 and Additional file 2: Figure S5). Furthermore, no transcripts of these remnant exons could be detected. Interestingly, all neognath birds share the deletion of exon 3. These results lead us to the conclusion that *stk33* became a unitary pseudogene in the evolutionary history of the class Aves during the late cretaceous period about 100 million years ago, at the branching point of Palaeognathae and Neognathae that was accompanied by an elevated rate of genomic rearrangements in neognaths [27, 29, 30]. We hypothesize that this pseudogenization of *stk33* might have become fixed in Neognathae due to either genetic redundancy or a non-orthologous gene displacement (NOD). Rho-kinase and protein kinase A have been identified as the most promising candidate genes by comparing their vimentin phosphorylation sites to those of STK33.

## Methods

### Animal material

All procedures concerning animals were performed in accordance with the published Directive 2010/63/EU of the European Parliament under a protocol approved by the local Administration District Official Committee. Moreover, all efforts were made to minimize the number of animals and their suffering.

Tissues expected to have high *stk33* expression were obtained from three bird species: zebra finch (*Taeniopygia guttata*), chicken (*Gallus gallus*) and African ostrich (*Struthio camelus*).

Adult male zebra finch (*Taeniopygia guttata*) were obtained from a local pet store, anaesthetized with carbon dioxide, killed by cervical dislocation and used for the preparation of brain and testis. Fertilized chicken (*Gallus gallus*) eggs were obtained from a local breeder and incubated for 6–17 days at 38 °C. After incubation eggs were opened and either whole embryos or the embryonic brain were isolated. Testes and complete heads from the African ostrich (*Struthio camelus*) were obtained from a local breeder. Ostrich heads were cut in half for preparation of the Cerebellum. After preparation all material was stored at −80 °C until further use.

### Sample preparation and sequencing

Tissue material was homogenized using a syringe and cannula. RNA isolation was performed using the RNeasy Mini Kit (Qiagen, Hilden, Germany), High Pure RNA Tissue Kit (Roche, Penzberg, Germany), PureLink® RNA Mini Kit (Life Technologies, Carlsbad, USA) or the GTC-method [81]. Subsequently, the RNA quality was tested using the 2100 Bioanalyzer (Agilent, Santa Clara, USA). DNA was isolated with the peqGOLD Tissue DNA Mini Kit (PeqLab, Erlangen, Germany).

1.5 μg of total RNA isolated from ostrich cerebellum and testis were each used for the library preparation using the TruSeq RNA Sample PrepV2 Kit (Illumina, San Diego, USA) and a 100 bp paired-end run was performed on an Illumina HiSeq 2000 (IMSB, Mainz, Germany). For zebra finch adult brain RNA sequencing, library preparation was carried out by GENterprise Genomics (Mainz, Germany) using 1.5 μg of total RNA and samples were sequenced in a 150 bp paired-end run on an Illumina HiSeq 2500 (IMSB, Mainz, Germany).

### Handling of SRA data

Genomic and transcriptomic data was downloaded from the NCBI SRA archive (http://www.ncbi.nlm.nih.gov/sra) and converted to fastq.gz files using the SRA Toolkit v2.3.5-2 [25]. An overview of all SRA files used in this study is shown in Table 2. The fastq.gz files were imported into CLC Genomics Workbench version 6.5.1 (CLC bio, Aarhus, Denmark) and trimmed as described below.

**Table 2:** Overview of all next generation sequencing data sets used in this study. SRA studies SRX736627, SRX738984 and SRX738987 were carried out at the IMSB Mainz. All other sequencing data was downloaded from the NCBI Sequence Read Archive

Organism	Source	Tissue	SRA study	Platform	Read count	Read layout
African ostrich (*Struthio camelus*)	transcriptomic	cerebellum	SRX736627	Illumina HiSeq 2000	70,355,488	100 bp paired
African ostrich (*Struthio camelus*)	transcriptomic	testis	SRX738984	Illumina HiSeq 2000	32,268,702	100 bp paired
African ostrich (*Struthio camelus*)	genomic	-	SRX334065	Illumina HiSeq 2000	210,096,016	100 bp paired
			SRX334066		237,908,622	
			SRX334067		183,703,328	
			SRX334068		170,644,024	
			SRX334072		160,894,606	
Emu (*Dromaius novaehollandiae*)	genomic	brain	SRP019803	Illumina HiSeq 2000	68,379,320	80 bp paired
Emu (*Dromaius novaehollandiae*)	transcriptomic	embryo	SRP019802	Illumina HiSeq 2000	51,118,472	80 bp paired
Emu (*Dromaius novaehollandiae*)	transcriptomic	generic sample	SRP001362	LS454	182,922	274 bp single
White-throated sparrow (*Zonotrichia albicollis*)	transcriptomic	brain	SRP029385	Illumina HiSeq 2000	196,980,056	100 bp single
Chicken (*Gallus gallus*)	transcriptomic	embryo	DRP000595	Illumina HiSeq 2000	461,468,546	100 bp single
Chicken (*Gallus gallus*)	transcriptomic	brain	SRX196389	Illumina HiSeq 2000	54,177,792	35 bp paired
			SRX196371		235,457,560	80 bp paired
			SRX196380		64,532,328	40 bp paired
Chicken (*Gallus gallus*)	transcriptomic	testis	SRX196397	Illumina HiSeq 2000	74,846,788	40 bp paired
			SRX196388		22,373296	36 bp paired
			SRX196379		231,583,782	80 bp paired
Chicken (*Gallus gallus*)	transcriptomic	hypo-thalamus	SRX316899	Illumina HiSeq 2000	191,561,330	100 bp paired
			SRX316900		188,549,958	
Collared flycatcher (*Ficedula albicollis*)	transcriptomic	embryo	ERX144565	Illumina Genome Analyzer IIx	49,600,302	100 bp paired
			-			
			ERX144572			
Japanese quail (*Coturnix japonica*)	genomic	-	DRX001717	Illumina HiSeq 2000	865,334,134	100 bp paired
Zebra finch (*Taeniopygia guttata*)	transcriptomic	brain	SRX738987	Illumina HiSeq 2500	9,535,066	150 bp paired

### Trimming of Illumina data

Sequencing reads were trimmed for quality and adapter sequences using CLC Genomics Workbench (CLC bio, Aarhus, Denmark). Quality trimming was done using an error probability limit of 0.01 and a maximum of 1 ambiguous base. All sequences below a read length of 15 bp were discarded.

### De novo assemblies and mappings

De novo assemblies of genomic and transcriptomic reads were conducted in CLC Genomics Workbench (CLC bio, Aarhus, Denmark) using standard parameters. *Stk33* contigs were identified using local BLASTn/BLASTx searches against a *stk33* database containing all available *stk33* sequences.

The feature “map reads to reference” of the CLC Genomics Workbench (CLC bio, Aarhus, Denmark) was used to map transcriptomic reads to transcriptomic contigs and “large gap read mapping” was employed to map transcriptomic data to genomic contigs. For intraspecies mappings a similarity of 98 % for at least 95 % of the read length was required, for interspecies mappings this similarity was reduced to 80 % similarity across 80 % of the read length. Mismatches were given a penalty of 2 and insertions or deletions were given a penalty of 3.

### Genomic studies

*Stk33* is located in a highly syntenic region spanning from *st5* to *ric3* (Additional file 1: Figure S1). In order to obtain the genomic target regions for *stk33* of the different birds we downloaded the annotated genomic contigs containing the region between genes *st5* and *tub* from UniGene. If these were not available we downloaded all assembled genomic contigs of the respective genome assembly project from the NCBI genome database (Additional file 1: Table S1) and identified contigs located in the *stk33* target region by local BLASTn searches with the genomic *stk33* contig from ostrich as reference. Putative exons of *stk33* were identified by comparison with the ostrich exons. Premature stop codons were identified by local BLASTx searches of single exons with the full length STK33 protein sequence from ostrich as reference.

### PCR and Sanger sequencing

cDNA was generated employing SuperScript® III reverse transcriptase (Invitrogen, Carlsbad, USA). Traditional PCRs were carried out using GoTaq® Polymerase (Promega, Fitchburg, USA) or Q5® High-Fidelity DNA Polymerase (NEB, Ipswich, USA). Takara LA Taq® DNA Polymerase (Takara, Otsu, Japan) was used for long Range PCRs. Primers were purchased from Invitrogen (Carlsbad, USA) and Sigma Aldrich (St. Louis, USA). DNA was denatured at 98 °C for 30 s, annealing was carried out at 60 °C for 30 s and extension was performed at 72 °C with an extension time of 1 min/kb. RACE PCRs were done using the GeneRacer™ Kit (Invitrogen, Carlsbad, USA). Sanger sequencing was carried out by StarSeq (Mainz, Germany).

### Availability of supporting data

All sequencing data generated in this study is available from the SRA-Archive (http://www.ncbi.nlm.nih.gov/sra) under the accessions numbers SRX738987 for the zebra finch, SRX738984 and SRX736627 for the African ostrich. *Stk33* sequences generated have been deposited into the GenBank database (http://www.ncbi.nlm.nih.gov/genbank/). Accession numbers are available from Additional file 1: Table S2. Due to GenBank limitations all sequences have also been included in Additional file 3.

## Additional files

Additional file 1:
**Contains the following items:** **Table S1** **All genomic contigs used in this study.** **Table S2** All birds examined in this study and their GenBank accessions numbers. **Figure S1** Representation of the syntenic genomic locus containing *stk33* in human, mouse and the chicken. **Figure S1** Phylogenetic tree of the class Aves with indications of analyzed bird species. **Figure S2** Phylogeny for STK33. **Figure S3** Alignment of the vimentin amino-terminal head domain from different bird species with human and mouse. **Figure S4** Alignment of the vimentin amino-terminal head domain from different bird species with human and mouse. Additional file 2: Figure S5. Sequence alignments of identified *stk33* exons of remaining neognaths to African ostrich *stk33* mRNA. Additional file 3:
**Contains the** ***stk33*** **sequences from all birds examined in this study.**
