# Supplementary material for: The serine/threonine kinase 33 is present and expressed in palaeognath birds but has become a unitary pseudogene in neognaths about 100 million years ago
Source: BMC Genomics. 2015 Jul 22;16(1):543. doi: 10.1186/s12864-015-1769-9 (PMC4509753; doi:10.1186/s12864-015-1769-9)
Supplement: Additional file 1: — Contains the following items: Table S1 All genomic contigs used in this study. Table S2 All birds examined in this study and their GenBank accessions numbers. Figure S1 Representation of the syntenic genomic locus containing stk33 in human, mouse and the chicken. Figure S1 Phylogenetic tree of the class Aves with indications of analyzed bird species. Figure S2 Phylogeny for STK33. Figure S3 Alignment of the vimentin amino-terminal head domain from different bird species with human and mouse. Figure S4 Alignment of the vimentin amino-terminal head domain from different bird species with human and mouse. [file 12864_2015_1769_MOESM1_ESM.pdf]

Supporting Information for “The serine/threonine kinase 33 is present and expressed in palaeognath birds but has become a unitary pseudogene in neognaths about 100 million years ago”

Table S1 All genomic contigs used in this study.

Organism	BioProject	Platform	Number of Contigs	Genome coverage
White-throated-tinamou (*Tinamus guttatus*)	PRJNA212876	Illumina HiSeq	142,367	100 x
Mallard (*Anas platyrhynchos*)	PRJNA46621	Solexa	227,448	60 x
Japanese quail (*Coturnix japonica*)	PRJDB1146	Illumina HiSeq	528,405	172 x
Chicken (*Gallus gallus*)	PRJNA13342	Sanger	27,102	12 x
Turkey (*Meleagris gallopavo*)	PRJNA42129	454-Titanium	152,913	17 x
Chuck-will’s widow (*Caprimulgus carolinensis*)	PRJNA212888	Illumina HiSeq	126,789	30 x
Sunbittern (*Eurypygia helias*)	PRJNA212893	Illumina HiSeq	115,170	33 x
Anna’s hummingbird (*Calypte anna*)	PRJNA212866	Illumina HiSeq	124,820	110 x
White-tailed tropicbird (*Phaethon lepturus*)	PRJNA212902	Illumina HiSeq	132,071	39 x
Rock pigeon (*Columba livia*)	PRJNA167554	Illumina HiSeq	100,098	60 x
Northern fulmar (*Fulmarus glacialis*)	PRJNA212894	Illumina HiSeq	112,214	33 x
Little egret (*Egretta garzetta)*	PRJNA232959	Illumina HiSeq	100,661	74 x
Red-throated loon (*Gavia stellata*)	PRJNA212895	Illumina HiSeq	126,712	33 x
Killdeer (*Charadrius vociferus*)	PRJNA212867	Illumina HiSeq	79,162	100 x
Bar-tailed trogon (*Apaloderma vittatum*)	PRJNA212878	Illumina HiSeq	105,649	28 x
Bald eagle (*Haliaeetus leucocephalus)*	PRJNA237821	Illumina HiSeq	31,786	103 x
Speckled mousebird (*Colius striatus*)	PRJNA212892	Illumina HiSeq	114,650	27 x
Red-legged seriema (*Cariama cristata*)	PRJNA212889	Illumina HiSeq	112,704	24 x
Saker falcon (*Falco cherrug*)	PRJNA168071	Illumina HiSeq	75,898	147 x
Peregrine falcon (*Falco peregrinus*)	PRJNA159791	Illumina HiSeq	83,080	138 x
Kea (*Nestor notabilis*)	PRJNA212900	Illumina HiSeq	93,561	32 x
Budgerigar (*Melopsittacus undulatus*)	PRJNA72527	Illumina HiSeq<br>454	70,862	7 x<br>16 x
Golden-collared manakin (*Manacus vitellinus*)	PRJNA212872	Illumina HiSeq	138,155	110 x
American crow (*Corvus brachyrhynchos*)	PRJNA212869	Illumina HiSeq	89,646	80 x
Tibetian ground tit (*Pseudopodoces humilis)*	PRJNA175930	Illumina HiSeq	27,051	96 x
Collared flycatcher (*Ficedula albicollis)*	PRJNA75089	Illumina GAII<br>Illumina HiSeq	30,842	60 x
Zebra finch (*Taeniopygia guttata)*	PRJNA17289	Sanger	124,805	5 x
White-throated sparrow (*Zonotrichia albicollis)*	PRJNA197293	Illumina HiSeq	37,661	63 x
Medium ground finch (*Geospiza fortis)*	PRJNA156703	Illumina HiSeq	95,828	115 x

Table S2 All birds examined in this study and their GenBank accessions numbers.

Common name	Scientific name	Accession number
Ostrich	*Struthio camelus*	KP072780
Emu	*Dromaius novaehollandiae*	-
White-throated-tinamou	*Tinamus guttatus*	BK008887
Anna's hummingbird	*Calypte anna*	BK009112
Chicken	*Gallus gallus*	BK009113
Rock dove	*Columba livia*	BK009114 - BK009116
Sunbittern	*Eurypygia helias*	BK009117 - BK009118
Wild turkey	*Meleagris gallopavo*	BK009119 - BK009120
Mallard	*Anas platyrhynchos*	BK009121 - BK009123
Little egret	*Egretta garzetta*	BK009124 - BK009126
Saker falcon	*Falco cherrug*	BK009127 - BK009129
Peregrine falcon	*Falco peregrinus*	BK009130 - BK009131
Budgerigar	*Melopsittacus undulatus*	BK009132
White-throated sparrow	*Zonotrichia albicollis*	BK009133
Ground tit	*Pseudopodoces humilis*	BK009134
Medium ground finch	*Geospiza fortis*	BK009135 - BK009137
American crow	*Corvus brachyrhynchos*	BK009138 - BK009141
Killdeer	*Charadrius vociferus*	BK009142 - BK009143
Speckled mousebird	*Colius striatus*	BK009144 - BK009146
Red-legged seriema	*Cariama cristata*	BK009147 - BK009148
Bald eagle	*Haliaeetus leucocephalus*	BK009149
Bar-tailed trogon	*Apaloderma vittatum*	BK009150
Chuck-will's widow	*Caprimulgus carolinensis*	BK009151 - BK009154
White-tailed tropicbird	*Phaethon lepturus*	BK009155 - BK009156
Golden-collared manakin	*Manacus vitellinus*	BK009157 - BK009158
Red-throated loon	*Gavia stellata*	BK009159 - BK009160
Japanese quail	*Coturnix japonica*	-
Zebra finch	*Taeniopygia guttata*	BK009161 - BK009162
Northern fulmar	*Fulmarus glacialis*	BK009163 - BK009167
Kea	*Nestor notabilis*	BK009168 - BK009171
Collared flycatcher	*Ficedula albicollis*	BK009172

Additional Figures

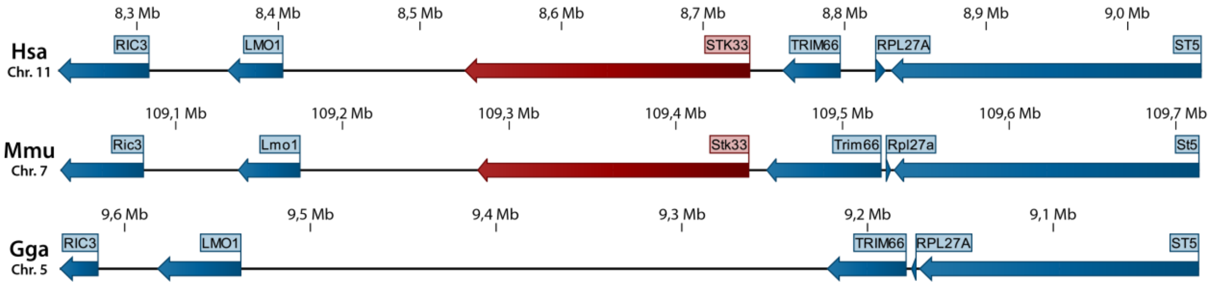

Figure S 1 Representation of the syntenic genomic locus containing *stk33* in human (Hsa), mouse (Mmu) and the chicken (Gga). The order and orientation of the genes in this locus is highly conserved between human, mouse and chicken. Only *rpl27a* is inverted in the chicken. There is no annotation for *stk33* between *trim66* and *lmo1* in the chicken.

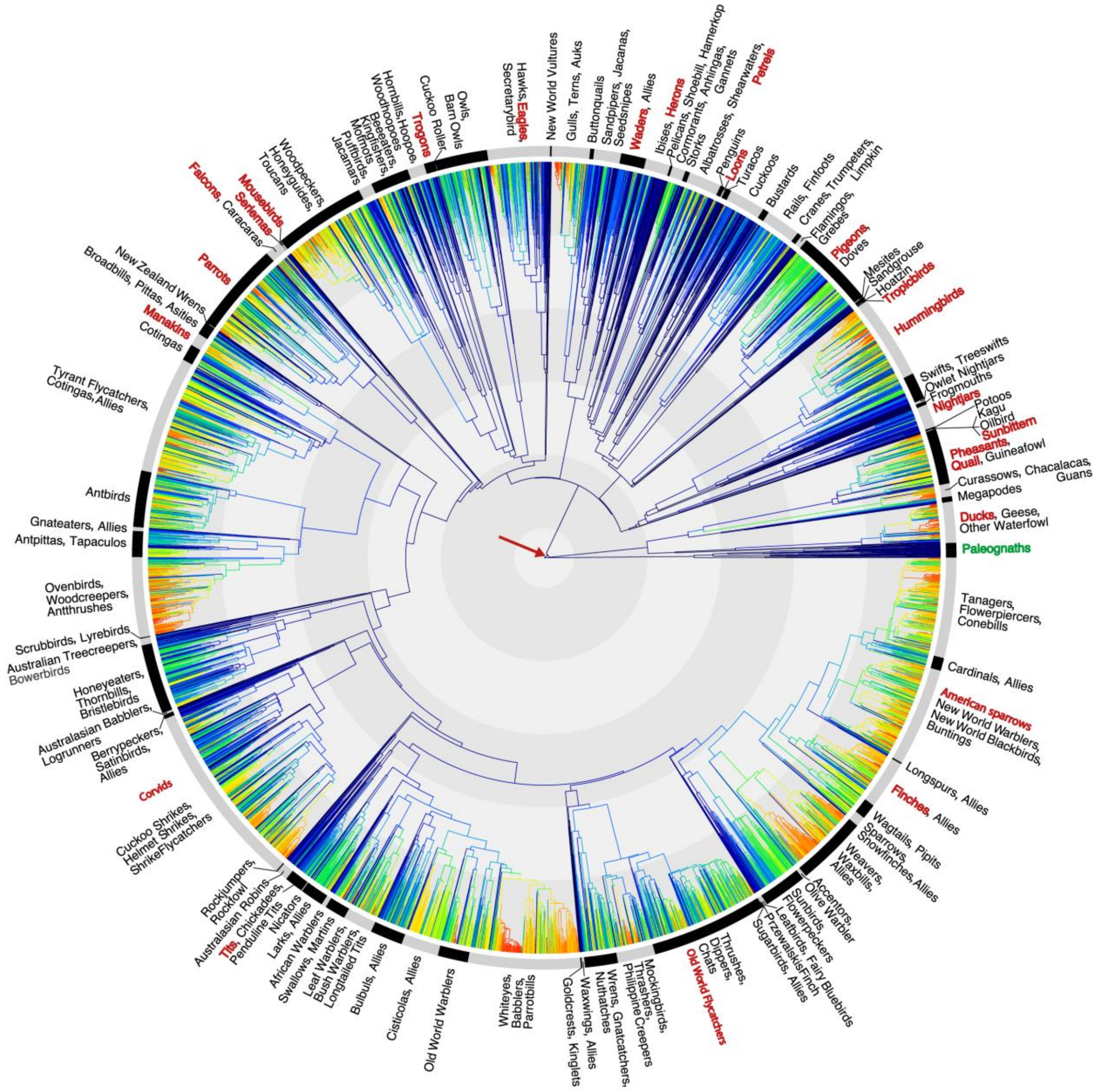

Figure S 2 Phylogenetic tree of the class Aves with indications of analyzed bird species. Families/Subclasses with a functional *stk33* are highlighted in green, families with only non-functional remnants of *stk33* are highlighted in red. The loss of *stk33* at the paleognath-neognath branch point is indicated by a red arrow. (Figure taken and adapted from birdtree.org with permission from the authors, including the main figure creator Gavin Thomas. Figure based on data from: Jetz, W; Thomas, GH; Joy, JB; Hartmann, K; Mooers, AO: The global diversity of birds in space and time. Nature 2012, 7424:444–448)

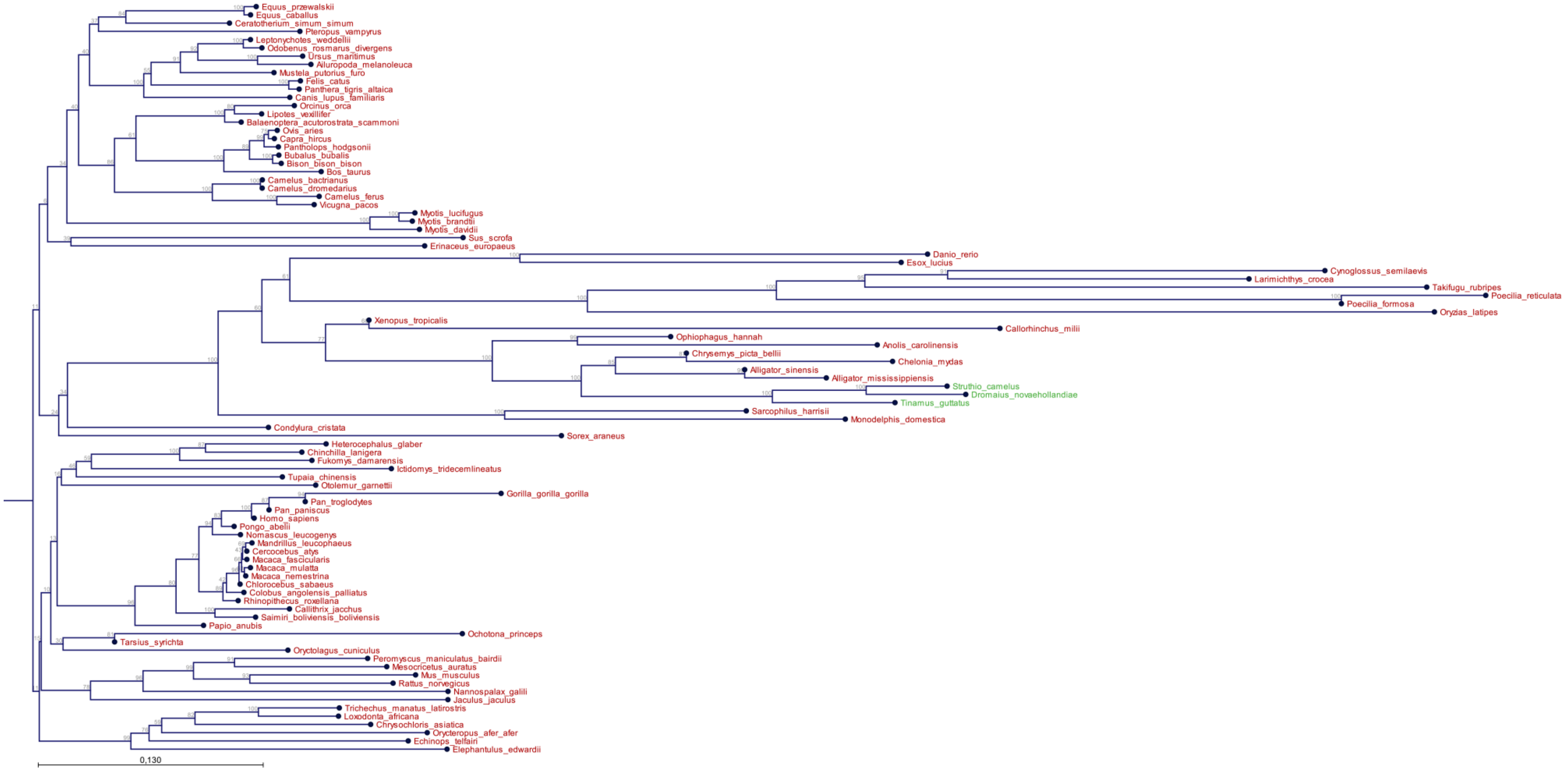

Figure S 3 Phylogeny for STK33. All supported STK33 proteins have been aligned and a maximum-likelihood-phylogeny has been built using the neighbor-joining algorithm. STK33 proteins from birds are highlighted in green. Bootstrap values are shown next to the corresponding node.

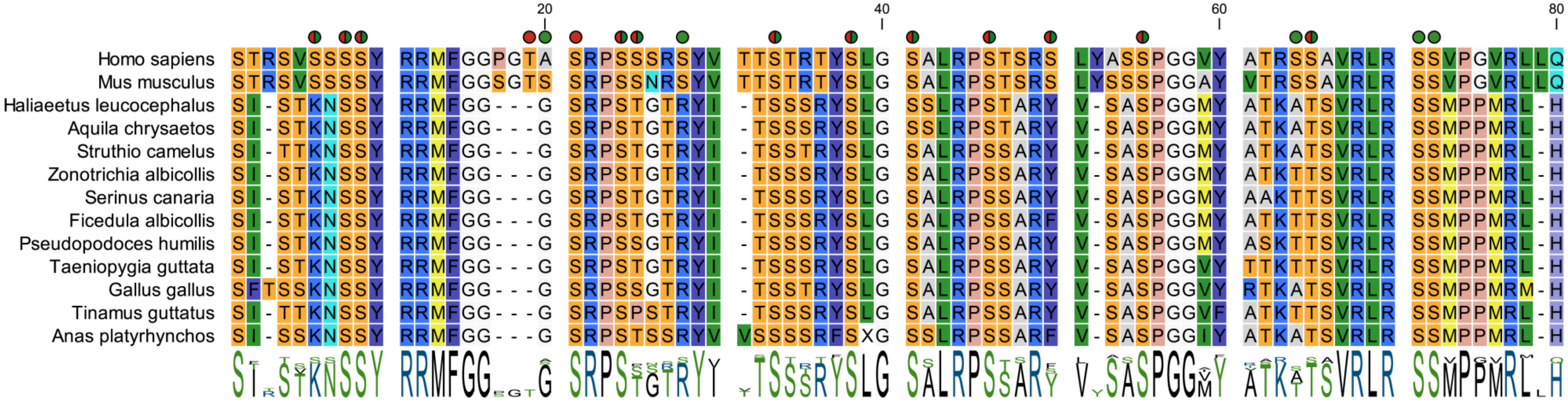

Figure S 4 Alignment of the vimentin amino-terminal head domain from different bird species with human and mouse. Phosphorylation sites from human [1;2] are indicated by red dots, phosphorylation sites from mouse [3] by green dots. Phosphorylation sites that have been described in both human and mouse are highlighted by green and red semicircles.

References

1. Herrmann, H; Aebi, U: **Structure, assembly, and dynamics of intermediate filaments**. *Sub-cellular biochemistry* 1998319–362.

2. Kochin, V; Imanishi, SY; Eriksson, JE: **Fast track to a phosphoprotein sketch - MALDI-TOF characterization of TLC-based tryptic phosphopeptide maps at femtomolar detection sensitivity**. *Proteomics* 2006, **21:**5676–5682.

3. Izawa, I; Inagaki, M: **Regulatory mechanisms and functions of intermediate filaments: a study using site- and phosphorylation state-specific antibodies**. *Cancer science* 2006, **3:**167–174.
