# Supplementary figures and images for: The serine/threonine kinase 33 is present and expressed in palaeognath birds but has become a unitary pseudogene in neognaths about 100 million years ago
Source: BMC Genomics. 2015 Jul 22;16(1):543. doi: 10.1186/s12864-015-1769-9 (PMC4509753; doi:10.1186/s12864-015-1769-9)

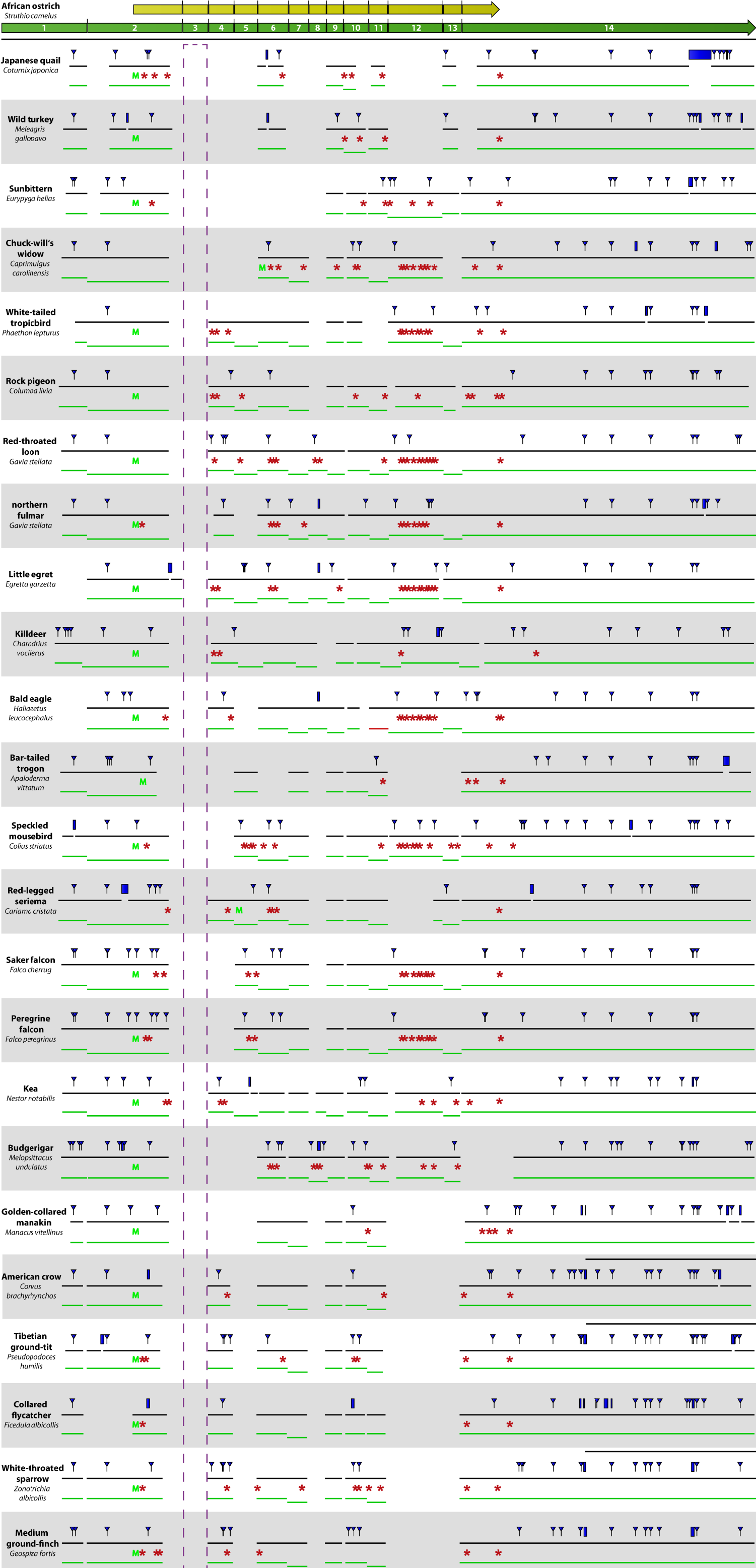

Supplement: Additional file 2: Figure S5. — Sequence alignments of identified stk33 exons of remaining neognaths to African ostrich stk33 mRNA. [file 12864_2015_1769_MOESM2_ESM.pdf]
